# Eradication of Ventricular Assist Device Driveline Infection in Paediatric Patients with Taurolidine

**DOI:** 10.3390/jcdd9010018

**Published:** 2022-01-10

**Authors:** Johannes Weichsel, Benito Baldauf, Hendrik Bonnemeier, Ernest W. Lau, Sven Dittrich, Robert Cesnjevar

**Affiliations:** 1 Department of Paediatric Cardiology, University Hospital Erlangen, 91054 Erlangen, Germany; johannes.weichsel@gmx.de (J.W.); sven.dittrich@uk-erlangen.de (S.D.); 2 Medical Faculty, Christian-Albrechts University Kiel, 24118 Kiel, Germany; bonnemeier@t-online.de; 3 Department of Cardiology, Royal Victoria Hospital, Grosvenor Road, Belfast BT12 6BA, UK; ernest.lau@btinternet.com; 4 Department of Paediatric Cardiothoracic Surgery, University Hospital Erlangen, 91054 Erlangen, Germany; robert.cesnjevar@kispi.uzh.ch; 5 Department of Paediatric Cardiothoracic Surgery, University Hospital Zürich, 8032 Zürich, Switzerland

**Keywords:** left ventricular assist device, infection, driveline, taurolidine

## Abstract

Ventricular assist devices (VADs) are used to provide mechanical circulatory support to patients with end-stage heart failure. The driveline connecting the external power source to the pump(s) of the intra-corporal VAD breaches the protective skin barrier and provides a track for microbes to invade the interior of the patient’s body. Driveline infection constitutes a major and potentially fatal vulnerability of VAD therapy. Driveline infection cannot traditionally be salvaged and requires the extraction of the entire VAD system. We report here the successful eradication of a VAD driveline infection with a taurolidine-containing antimicrobial solution used for preventing the infection of cardiac implantable electronic devices. If replicated in more cases, the novel treatment concept described here may provide a valuable alternative management strategy of salvage rather than explantation for VAD driveline infection.

## 1. Introduction

Ventricular assist devices (VADs) provide mechanical circulatory support to patients with end-stage heart failure, and may be used as either a bridge to cardiac transplantation or a destination therapy in their own right. VADs can be intra- or extra-corporal, depending on whether the pump(s) driving the circulation of blood are located inside or outside of the patient’s body. While technical advances have greatly improved the safety and reliability of these highly sophisticated electro-mechanical devices, the rates of certain complications (e.g., thrombosis, infection) have remained steady. Infection of the driveline connecting the external power source to the pump(s) inside the patient’s body is the leading cause of hospital re-admission for intracorporeal VADs. Mortality is 5.6 times higher in patients with VAD-related infections and can exceed 35%. Microbes form biofilms on the driveline and can be resistant to antimicrobial agents commonly used during VAD revision [1,2].

TauroPace™ (TauroPharm GmbH, Waldbüttelbrunn, Bavaria, Germany) is an antimicrobial solution used for preventing infection from cardiac implantable electronic devices (CIEDs). The agent is versatile in its application and can be used for wiping the external surfaces of medical devices, flushing their inner surfaces (if they have lumens), or irrigating the surgical site. Its main active ingredient is Taurolidine, an amino acid compound that breaks down by hydrolysis in vivo to release methylol groups. Methylol groups readily react with and denature the peptidoglycans in the bacterial cell wall, and the polysaccharide and lipopolysaccharide components of endotoxins and certain susceptible exotoxins. The results are the destruction of the bacterial cell, the inhibition of surface attachment biofilm formation, and the inactivation of endotoxins and some exotoxins. The active taurolidine compounds have a half-life of 1.56 h and 6 h in the presence of saccharides, peptides and glycans. The final products of taurolidine metabolism are the non-essential amino acid taurine, and water. Taurine degradation leads to an acidic environment in the surgical site, which increases the chemical activities of the Taurolidine compound, setting up a positive feedback loop for its biological effects [3]. Taurine has been shown to promote wound healing in vitro, in animal models, and in vivo.

## 2. Patients

Both minors were initially implanted with the Heart Ware™ (Medtronic, Minneapolis, MN, USA) left ventricular assist device (LVAD) in the paediatric cardio-thoracic department of the University Hospital Erlangen.

The first patient received the LVAD as a destination therapy for terminal heart failure in dilated cardiomyopathy. The patient was not eligible for urgent heart transplantation due to a rare mitochondriopathy. The second patient received the LVAD as a bridge to cardiac transplantation. The underlying pathology was dilated cardiomyopathy secondary to chronic non-viral myocarditis, incidentally discovered when the patient was investigated for a thromboembolic stroke in the middle cerebral artery and bilateral pneumonia.

**Figure 1:**
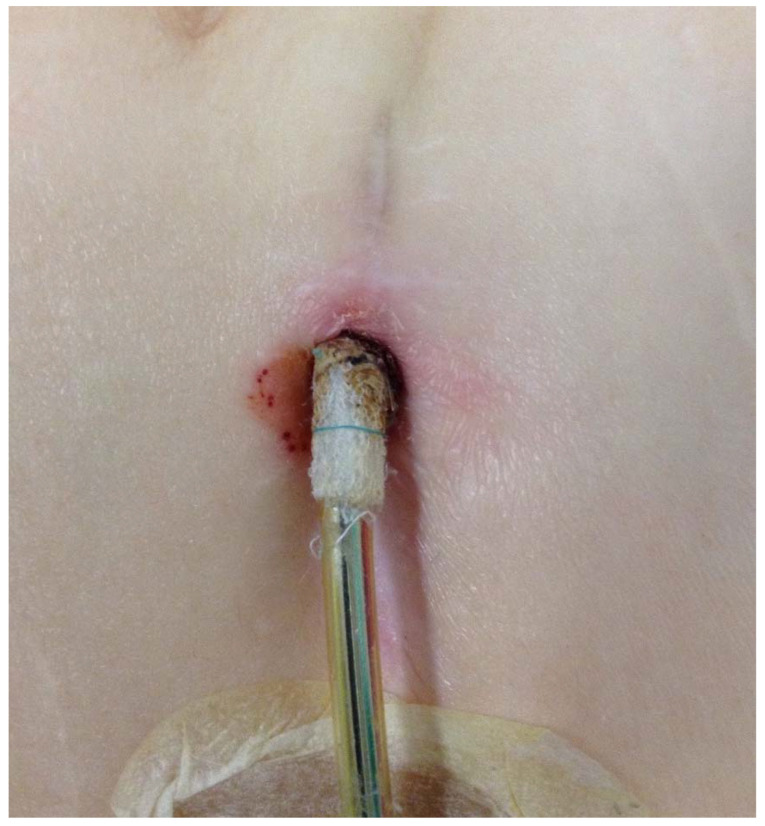
Typical appearance of petechial hemorrhages in the skin around the exit site in VAD driveline infection.

Both patients displayed the skin appearance typical of driveline infection when admitted to our centre (Figure 1). Endocarditis was ruled out in both cases. The patients were treated with antibiotics, first empirically and then adjusted according to the sensitivity from bacteriology testing (first patient: Staphylococcus aureus and Escherichia coli; second patient: Staphylococcus epidermidis). On the basis of the clinical suspicion of driveline infection, surgical revision of the LVAD system was planned.

VAD salvage procedure: The skin around the exit site and the fibrous tissues around the driveline were excised. The driveline was mobilised from the subcutaneous tissues and washed with the Taurolidine solution. The infected, partially necrotic wound bed was debrided until healthy uninfected tissues were seen. The driveline was brushed to remove any adherent debris. The entire surgical field from the exit site of the driveline to the mediastinum was thoroughly irrigated with the Taurolidine solution (250 cc in the first case, 200 cc in the second case). The knitted Poly-ethylene-terephthalate velour cover surrounding the driveline was saturated with the Taurolidine solution, placed in the final position, and secured to the fascia rectus abdominis with Polyester sutures. The original skin exit site was reused and no new exit point for the driveline was fashioned. The subcutaneous space was closed with a Polyglactin suture. The skin edges were closed with interrupted non-absorbable monofilament sutures. Negative pressure therapy (Prevena™ Incision Management Therapy, 3M™, Maplewood, MN, USA) was applied to the surgical wound for 7 days, with the sponge dressing changed every 3 days. Aseptic wound dressing was then applied and changed daily for the next 7 days. Parenteral antibiotic therapy was administered for a total of 14 days in the first case and 12 days in the second case. Inflammation had clinically disappeared from the driveline skin exit site 3 days after the revision procedure, which we had not experienced before the use of the Taurolidine solution. VAD driveline infection did not reoccur after the 10-month follow-up (December 2021) and both patients remained in excellent condition.

## 3. Discussion and Conclusions

Our surgical approach for managing driveline infection should be “standard of care” and performed in identical or similar fashions in centres with the appropriate expertise. The use of various antiseptics to irrigate the surgical field in the revision of driveline infection may also be common. The use of Taurolidine in this context is novel. The use of Taurolidine to treat surgical infection in the operation room was first described in some anecdotal reports in the 1980s. Subsequently, Taurolidine has been most commonly used in locking solutions to prevent catheter-related bloodstream infection in fragile patients [4,5,6]. As far as we are aware, Taurolidine has not been used to treat a VAD driveline infection before. However, the positive initial experience with the use of Taurolidine for this purpose had led to its rapid adoption into routine clinical practice in our centres.

Despite the encouraging preliminary results with the use of the Taurolidine solution to treat driveline infection (and salvage the VAD system), further confirmation is needed before its widespread adoption can be recommended. There is extensive clinical experience with the use of the Taurolidine solution in CIED procedures, giving reassurance on the safety if, not the efficacy of the product.

## Data Availability

Please note that no data set was generated due to the nature of this publication.
